# miRNA profiling of NurOwn®: mesenchymal stem cells secreting neurotrophic factors

**DOI:** 10.1186/s13287-017-0692-1

**Published:** 2017-11-07

**Authors:** Yael Gothelf, Haggai Kaspi, Natalie Abramov, Revital Aricha

**Affiliations:** BrainStorm Cell Therapeutics Ltd., 12 Bazel St., POB 10019, Kiryat Arieh, Petach-Tikva 4900101 Israel

**Keywords:** Mesenchymal Stromal Cells, Neurotrophic Factors, Amyotrophic Lateral Sclerosis, MicroRNAs

## Abstract

**Background:**

MSC-NTF cells are Mesenchymal Stromal Cells (MSC) induced to express high levels of neurotrophic factors (NTFs) using a culture-medium based approach. MSC-NTF cells have been successfully studied in clinical trials for Amyotrophic Lateral Sclerosis (ALS) patients.

MicroRNAs (miRNA) are short non-coding RNA molecules that coordinate post-transcriptional regulation of multiple gene targets. The purpose of this study was to determine whether the miRNA profile could provide a tool for MSC-NTF cell characterization and to distinguish them from the matched MSC from which they are derived.

**Methods:**

NTF secretion in the culture supernatant of MSC-NTF cells was evaluated by ELISA assays. The Agilent microarray miRNA platform was used for pairwise comparisons of MSC-NTF cells to MSC. The differentially expressed miRNAs and putative mRNA targets were validated using qPCR analyses.

**Results:**

Principal component analysis revealed two distinct clusters based on cell type (MSC and MSC-NTFs). Nineteen miRNAs were found to be upregulated and 22 miRNAs were downregulated in MSC-NTF cells relative to the MSC cells of origin. Further validation of differentially expressed miRNAs confirmed that miR-3663 and miR-132 were increased 18.5- and 4.06-fold, respectively while hsa-miR-503 was reduced more than 15-fold, suggesting that miRNAs could form the basis of an MSC-NTF cell characterization assay. In an analysis of the miRNA mRNA targets, three mRNA targets of hsa-miR-132-3p (HN-1, RASA1 and KLH-L11) were found to be significantly downregulated.

**Conclusions:**

We have demonstrated that MSC-NTF cells can be distinguished from their MSCs of origin by a unique miRNA expression profile.

**Trial Registration:**

Clinicaltrial.gov identifier NCT01777646. Registered 12 December 2012.

## Background

Mesenchymal stromal cells (MSC) are multipotent cells capable of differentiating into cells of multiple lineages [1]. MSC are being evaluated as a potential therapy for a wide variety of degenerative and immunological disorders [2].

We have developed a culture-medium based method for inducing MSCs to secrete enhanced levels of multiple neurotrophic factors (NTFs) including glial-derived neurotrophic factor (GDNF), brain-derived neurotrophic factor (BDNF) vascular endothelial growth factor (VEGF) and hepatocyte growth factor (HGF) [3]. MSC-NTF cells (NurOwn®) have been successfully used in clinical trials for amyotrophic lateral sclerosis (ALS) patients [4].

NTFs are small, naturally occurring polypeptides that support the development and survival of neurons [5]. NTFs are potent survival factors for embryonic, neonatal, and adult neurons, and have been evaluated in various neurodegenerative disease clinical trials over the past 25 years [6, 7]. MSC-NTF cells that have been induced to enhanced secretion of NTFs, offer a novel method for simultaneously delivering multiple NTFs to patients with neurodegenerative diseases such as ALS, while leveraging the potential immunomodulatory therapeutic benefits of MSCs [8]. Furthermore, a recent study demonstrated potential therapeutic benefits of MSC-NTF cells in an animal model of autism [9].

ALS (also known as Lou Gehrig’s disease) is a rare, relentlessly progressive and lethal neurodegenerative disease. At the cellular level, ALS is characterized by the progressive degeneration of upper and lower motor neurons in the motor cortex, brainstem, and spinal cord leading to progressive functional impairment and ultimately death [10].

Three clinical studies with NurOwn® (the MSC-NTF cell therapy) in ALS patients have been completed. The first two open-label studies [4], confirmed that the treatment was safe and well tolerated either by the intrathecal (IT) or by the intramuscular (IM) route of administration as well as by combined IT and IM administration. These studies demonstrated preliminary indications of efficacy, by slowing the rate of disease progression, as measured by the ALS Functional Rating Scale-Revised (ALSFRS-R) score. The recently completed US phase 2 multicenter double-blind placebo-controlled study confirmed these preliminary findings and suggested that meaningful efficacy could be achieved following a single MSC-NTF cell transplantation in a subgroup of patients that excluded slow progressors (submitted for publication). These findings are to be confirmed in a larger repeat-dose multicenter US phase 3 program.

MicroRNAs (miRNAs) are short (17-24 nt), single-stranded, endogenous non-coding RNAs that regulate gene expression by post-transcriptional silencing and/or mRNA degradation and can play important regulatory roles in animals and plants by targeting mRNAs for cleavage or translational repression. miRNAs have been shown to play critical roles in several biological processes, including cell differentiation, cell development, cell growth and apoptosis, by regulating gene expression through either the inhibition of mRNA translation or the induction of mRNA degradation [11–13].

Single miRNA can target multiple mRNAs, overall targeting approximately 60% of human genes. They regulate multiple and diverse cellular pathways and processes in normal and disease situations leading to changes in cell phenotype.

miRNA act in concert with Argonaute proteins within the RISC complex to suppress translation in a manner which is dependent on incomplete Watson-Crick base pairing between the so-called ‘seed’ sequence of the miRNA with complementary sequences in the target gene, usually in the 3’-UTR region of the mature message.

This study aimed to characterize an MSC-NTF miRNA fingerprint by identifying miRNAs that are significantly differentially expressed in MSC-NTF cells compared to the donor-matched MSCs by selecting a panel of mi-RNAs to monitor cell differentiation and performance, to be used as release criteria, and as an *in-vivo* identification assay.

## Methods

### Cells

MSC were isolated from healthy volunteers (Lonza, Walkersville, MD, USA) and from ALS patients’ bone marrow and expanded in culture. ALS patients were consented in accordance with the Helsinki declaration in the context of the phase 2a clinical trial (Clinicaltrial.gov identifier NCT01777646). The study was approved by the ethics committee of the Hadassah Hebrew University Medical Center, Jerusalem, Israel, and by the Director General of the Israel Ministry of Health.

MSC-NTF cells were induced to differentiate from each of the MSC donors, using a culture medium-based approach as previously described [3]. Briefly, MSCs were induced to differentiate into MSC-NTF cells using a medium-based approach in which cells were incubated in medium containing 1 mM dibutyryl cyclic AMP (cAMP), 20 ng/ml human basic fibroblast growth factor (hbFGF), 5 ng/ml human platelet-derived growth factor (PDGF-AA), and 50 ng/ml human Heregulin β1.

### NTF secretion

NTF secretion was evaluated by ELISA for GDNF (DuoSet, R&D Systems, Minneapolis, MN, USA) VEGF and HGF (Quantikine, R&D Systems) in cell culture supernatant before and after MSC differentiation into MSC-NTF cells.

### Microarray profiling and validation

Total RNA was extracted from eight independent, matched donor bone marrow-derived MSC and derived MSC-NTF cells of healthy donors and ALS patients using the Cell & Plant miRCURY™ RNA isolation kit (Exiqon, Copenhagen, Denmark). All RNA samples had a RIN > 7.

Microarray analysis was performed on 100 ng total RNA using Agilent’s miRNA platform (SurePrint G3 Human v16 microRNA 8 × 60K microarray slides, Agilent Technologies, Cheadle, UK). Data pre-processing and normalization was carried out using the “AgiMicroRNA” package in Bioconductor (https://www.bioconductor.org/packages/devel/bioc/vignettes/AgiMicroRna/inst/doc/AgiMicroRna.pdf). miRNAs differentially expressed between the MSC-NTF and MSC cells were identified by fold change analysis (pFDR < 0.05, fold change > 1.5). Candidate miRNAs from microarray data for future normalization of quantitative reverse transcription (qRT)-PCR were identified using the two one-sided tests approach (pFDR < 0.05, fold change < 2.0).

Expression analysis of the differentially expressed mi-RNAs was carried out by qRT-PCR using miRCURY LNA™ Universal RT microRNA PCR (Exiqon) except for miR-3663 that was analyzed using a miScript assay (Qiagen, Hilden, Germany), and a Roche LightCycler 480 (Roche Diagnostics Ltd, Burgess Hill, UK). Identification of miRNAs for normalization of qRT-PCR was carried out using the GeNorm algorithm [14] as implemented in Biogazelle qbase + v2.5 (Biogazelle, Ghent, Belgium). Mean fold changes were determined between normalized relative expression values for MSC and MSC-NTF cells and tested for statistical significance using Student’s *t* test (*p* < 0.05).

### Target mRNA validation

For validating the mRNA targets of the validated miRNAs, qPCR was carried out using RT2 mRNA PCR (Qiagen, Hilden, Germany) and a Roche LightCycler 480 on the RNA samples of MSC and MSC-NTF cells of eight different donors (six ALS patients and two healthy donors).

## Results

### NTF secretion

**Fig. 1:**
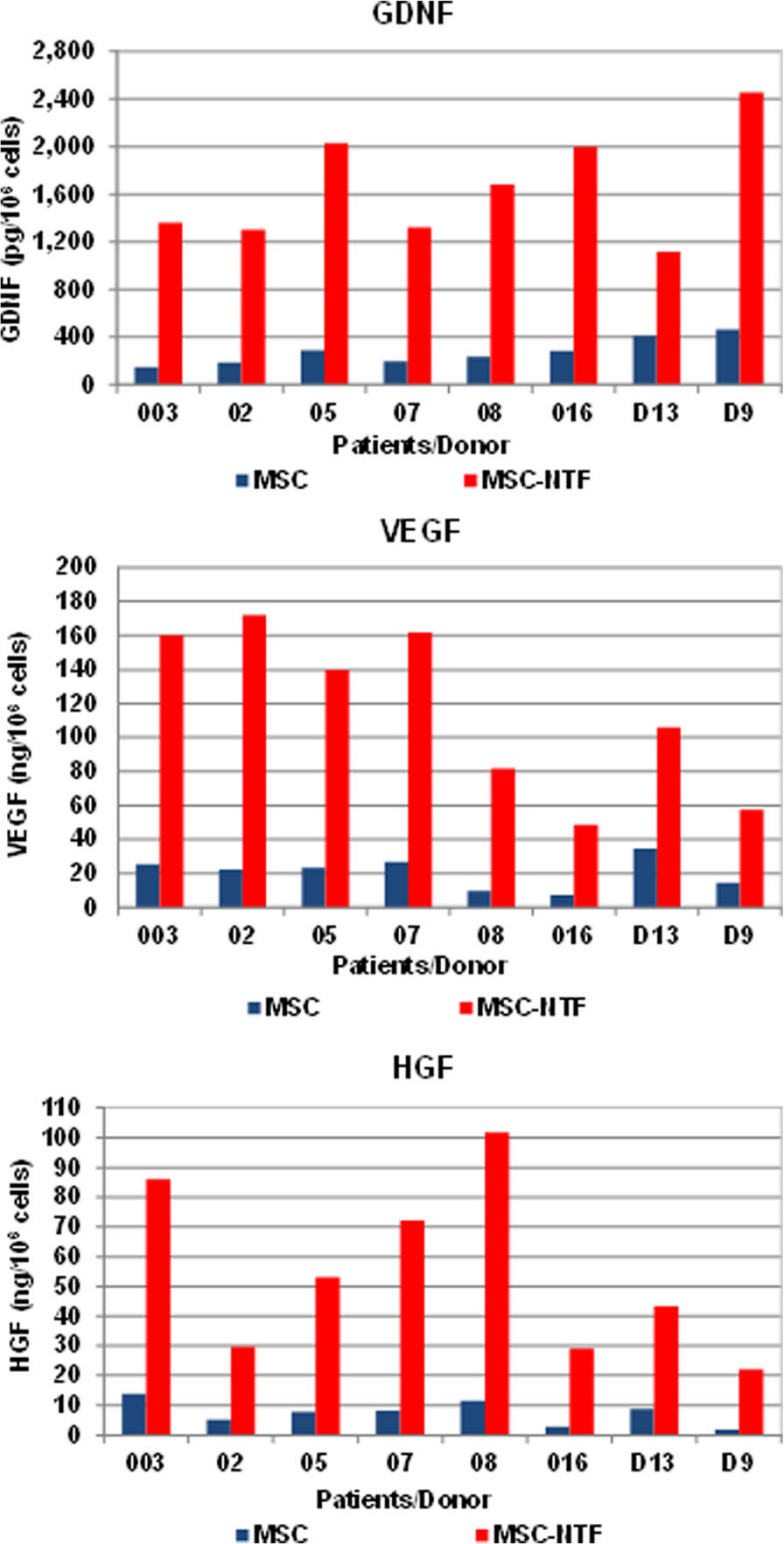
NTF secretion by MSC and MSC-NTF cells of the same patient/donor. MSC of six ALS patients and two healthy donors (D13 and D9) were induced to differentiate into MSC-NTF cells and secretion of neurotrophic factors GDNF, VEGF and HGF was measured in the culture supernatant by ELISA before and after differentiation. Average fold change MSC-NTF/MSC 6.56, 6.01, and 7.85 respectively (*p* < 0.005). *GDNF* glial-derived neurotrophic factor, *HGF* hepatocyte growth factor, *MSC* mesenchymal stromal cells, *NTF* neurotrophic factors, *VEGF* vascular endothelial growth factor

We induced MSCs to differentiate into MSC-NTF cells, using a culture medium-based process. The MSC-NTF cells maintained the MSC immunophenotype, whereby > 95% of the population expresses CD73, CD90 and CD105 as determined by flow cytometry analyses and secrete significantly higher levels of NTFs such as GDNF, VEGF and HGF versus naïve MSC (Fig. 1).

### miRNA profiling

Matched MSC and MSC-NTF cells samples from four different ALS patients (patient ID 02, 03, 05, and 07) were analyzed using the Agilent miRNA platform. A total of 160 miRNAs were reliably detected across all the samples analyzed (present in at least one sample). An average of 199.75 ± 22.72 and 227.75 ± 14.48 (mean ± SD) miRNAs were identified in the MSC and MSC-NTF cell populations respectively.

To gain an overview of the donor-to-donor variability within each cell group and the relationships between the different cell groups, a visualization of the complete dataset was produced by PCA using all 160 detected miRNAs. The PCA plot represents the information content (variance) of each complete microRNA-ome dataset on the plot, as a single point in the principal component (PC) projection. The key point is the similar datasets cluster together.

**Fig. 2:**
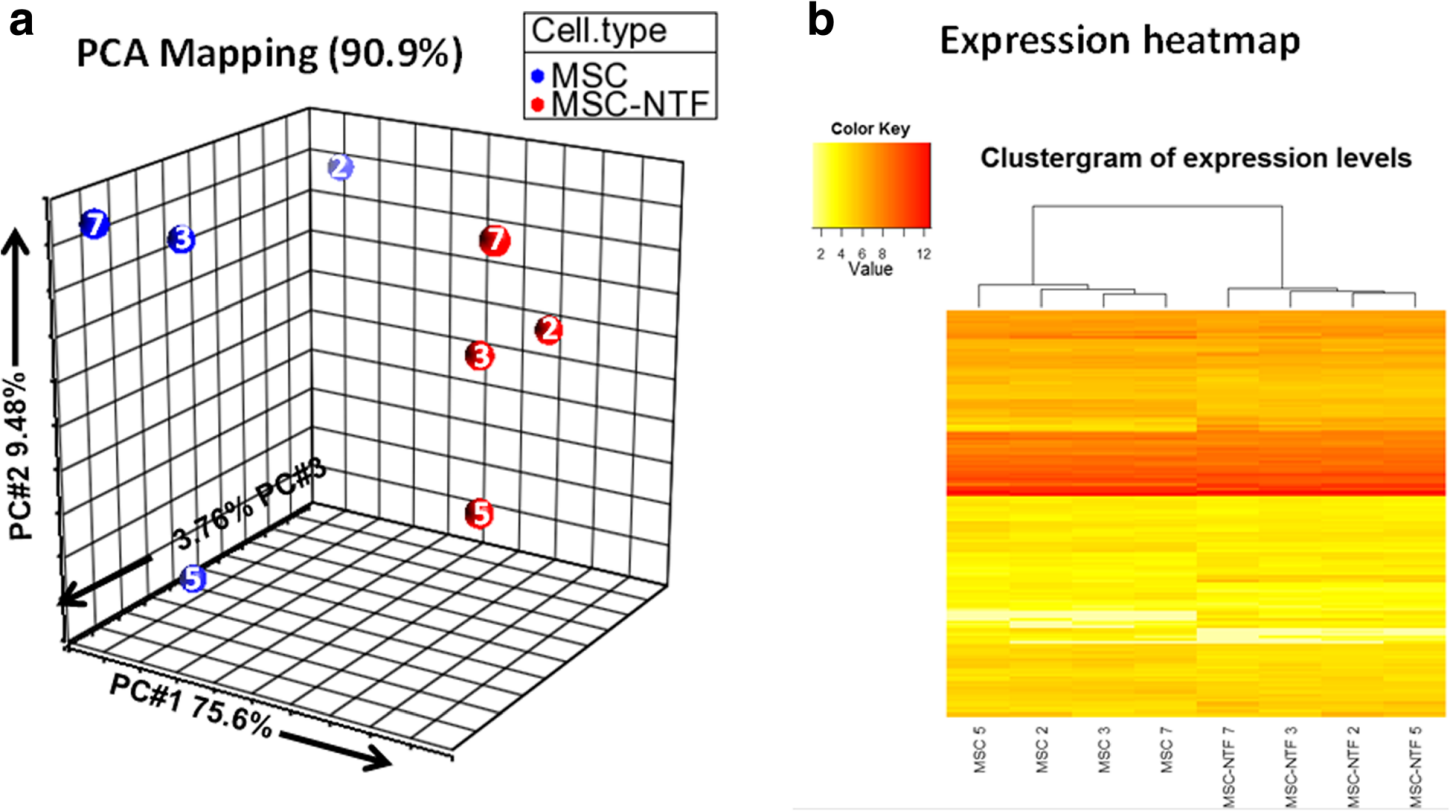
Comparison analysis of the MSC and MSC-NTF cell types based on all 160 detected miRNAs with cell type and donor ID indicated. **a** Representation of the eight cellular matched miRNA profiles of the four ALS patients in a 3D PCA projection, including donor ID (02, 03, 05, and 07); **b** Representation of the eight cellular miRNA profiles as a heatmap clustergram plot after hierarchical clustering, including donor ID. *MSC* mesenchymal stromal cells, *NTF* neurotrophic factors, *PCA* principal component analysis

This was initially done as a projection of the first three PCs (Fig. 2a). An alternative visualization of the expression patterns for the miRNAs in each sample and the sample relationships was generated using a heatmap based on agglomerative hierarchical clustering (Fig. 2b).

The PCA and heatmap clustergram of MSC and MSC-NTF cells show that the sample set clearly separate, forming two distinct clusters based on cell type.

### Analysis of miRNA

Forty-one different miRNAs were found to be differentially expressed in the MSC-NTF cells as compared to MSC. Eight miRNAs, equivalently expressed in the two cell types were identified, were used as candidate normalizers to support qPCR validation studies.

**Fig. 3:**
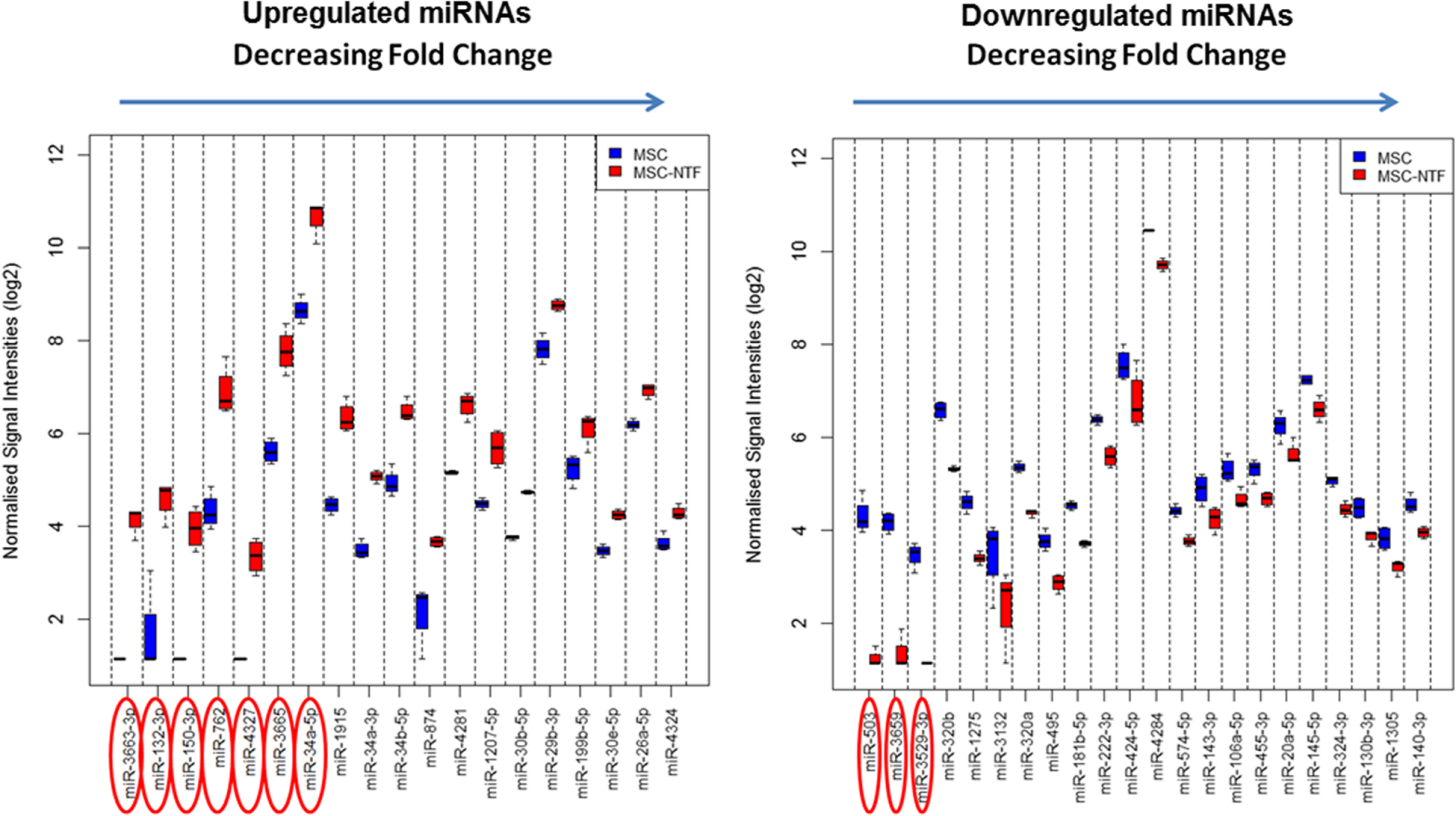
Differentially expressed miRNAs. Expression profiles of the 19 upregulated miRNAs (*left panel*) and the 22 downregulated miRNAs in MSC-NTF versus MSC on a log2 scale (*right panel*). The miRNAs most strongly down- and up-regulated in MSC-NTFs are highlighted with *red ovals*. When the expression of a miRNA was below the level of detection for the arrays, a nominal intensity value is given to these data points to avoid errors arising from non-computable mathematical operations during subsequent data analyses. *MSC* mesenchymal stromal cells, *NTF* neurotrophic factors

Statistical comparisons of the miRNA profiles of the differentially expressed miRNAs showed that 19 were upregulated in MSC-NTF versus MSC and 22 were downregulated in MSC-NTF versus MSC (Fig. 3).

### Gene ontology analysis of predicted target genes

The function of up- and downregulated miRNAs was studied using gene ontology analysis of predicted target genes. The analysis was performed on genes which were predicted to be miRNA targets by the miRNet database that integrates 11 different miRNA databases (http://www.mirnet.ca/faces/home.xhtml). To increase the level of reliability, only genes which were predicted to be targeted by at least three downregulated miRNAs or two upregulated miRNAs were included in the analysis (Table 1). While targets of upregulated miRNAs were found to be involved in promoting cell proliferation, targets of miRNAs which were downregulated were identified as genes which participate in neurogenesis, central nervous system development and cell cycle regulation (Table 1).

**Table 1:** Gene ontology analysis

GO biological process	No of genes	*p* value
Gene sets predicted to be upregulated following downregulation of at least three miRNAs (248 genes in analysis)		
Central nervous system development	31	5.15E-05
Regulation of cellular protein metabolic process	49	5.15E-05
Regulation of growth	25	5.15E-05
Cell division	24	5.15E-05
Regulation of cell cycle	34	5.15E-05
Regulation of translation	15	0.000127
Negative regulation of cellular metabolic process	48	0.000127
Negative regulation of apoptotic process	27	0.000127
Regulation of protein metabolic process	51	0.000127
Neurogenesis	27	0.000127
Gene sets predicted to be targeted (downregulated) by at least two upregulated miRNAs (164 genes in analysis)		
Gland development	13	0.00222
Positive regulation of cell proliferation	20	0.00701
Negative regulation of transcription from RNA polymerase II promoter	15	0.00831
Positive regulation of cell migration	10	0.00831
Regulation of chromosome organization	7	0.00831
Regulation of cell proliferation	28	0.00831
Negative regulation of apoptotic process	17	0.00831
Negative regulation of programmed cell death	17	0.00831
Positive regulation of developmental processes	19	0.00831
Positive regulation of cellular component organization	15	0.00831

*miRNA*: microRNAs

### Validation of differentially expressed miRNA

To validate the differentially expressed miRNAs, matched RNA samples of MSC and MSC-NTF cells of eight different donors (six ALS patients and two healthy donors) were analyzed. Of a panel of seven candidate-normalizing miRNAs tested, hsa-miR-19b-3p and hsa-miR-22-3p were selected as appropriate normalizers for this study.

The most differentially expressed miRNAs identified in the microarray analysis or those with a biologically relevant target gene function were chosen for validation by qRT-PCR (Table 2).

**Table 2:** qPCR validation of differentially expressed miRNAs

miRNA ID	Microarray fold change (MSC-NTF vs MSC)	Function	qPCR validated (*p* ≤ 0.05)	Fold change (MSC-NTF vs MSC)
hsa-miR-503-5p	−8.38	Anti-angiogenic [17, 25] Promotes osteogenesis [26] (Originates from the same primary RNA transcript as miR-424 [27]	Yes	−30.5
hsa-miR-320b	−2.41	Involved in neural development, anti-angiogenic [28]	Yes	−1.36
hsa-miR-320a	−1.97	Targets VEGF A [16]	No	−1.28
hsa-miR-222-3p	−1.74	Negative modulator of angiogenesis Inhibition of osteogenic differentiation [29]	No	−1.25
hsa-miR-424-5p	−1.72	Anti-angiogenic [25] Targets VEGF A, VEGFR-2 and FGF2 [15]	Yes	−1.94
hsa-miR-3663-3p	8.03	On/off signal. No validated mRNA target	Yes	18.5
hsa-miR-132-3p	7.85	Pro-angiogenic [30] Inhibits osteoblast differentiation [31] Regulation of synaptic structure [18]	Yes	4.06
hsa-miR-150-3p	6.99	On/off signal. Inhibits osteoblast differentiation downstream of TNF-α [32]	Not validated but significantly downregulated	−4.44
hsa-miR-762	5.91	Upregulated in neural precursors [33]	No	1.47
hsa-miR-34a-5p	4.05	Tumor suppressor: pro-apoptotic and anti-proliferative [34] TNF-α suppressor [35] Inhibits osteoblast differentiation [36]	Yes	3.16

*miRNA* microRNAs, *MSC* mesenchymal stromal cells, *NTF* neurotrophic factors, *VEGF(R)* vascular endothelial growth factor (receptor), *FGF* fibroblast growth factor, TNF-α tumor necrosis factor alpha

qRT-PCR validation confirmed that six (hsa-miR-503, hsa-miR-320b, hsa-miR-424-5p, hsa-miR-34a-5p hsa-miR-132-3p and hsa-miR-3663-3p) of the ten miRNAs that were analyzed were found to be differentially expressed (*p* < 0.05), while the differential expression of hsa-miR-320a, hsa-miR-222-3p and hsa-miR-762) was just outside the significance cutoff *p* value (Table 2).

**Fig. 4:**
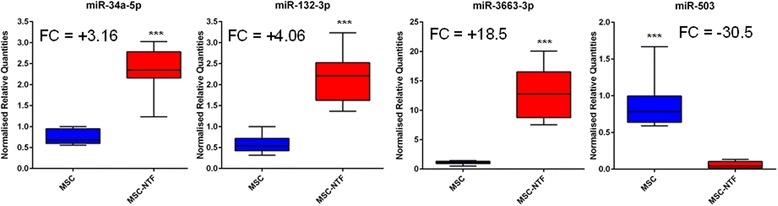
Validated differentially expressed miRNAs. Differential expression of miRNAs identified in the microarray was validated by qPCR analysis of MSC and MSC-NTF cells of eight different donors (six ALS patients and two healthy donors). ***p < 0.001, two-sided *t* test. *FC* fold change, *MSC* mesenchymal stromal cells, *NTF* neurotrophic factors

Of the differentially expressed miRNAs, hsa-miR-3663-3p was upregulated 18.5-fold and hsa-miR-503 was downregulated 30.5-fold (Fig. 4).

Non-specific amplification products were identified for miR-150-3p. miR-222-3p, miR-320a and 320b and miR-424-5p had low (and in some cases not significant) fold change between MSC and MSC-NTF cells limiting their diagnostic usefulness.

### Target mRNA expression

To further investigate the potential effect of differentially expressed miRNAs we looked at the putative mRNA targets of the identified miRNAs.

Of the most significantly differentially expressed miRNAs that were validated by qPCR, miR-3663 has no known mRNA targets. We therefore further investigated the mRNA targets of hsa-miR-132-3p and hsa-miR-503, which were confirmed to be upregulated 4-fold and downregulated 30-fold, respectively.

Putative validated mRNA targets of hsa-miR-132-3p and miRNA hsa-miR-503-5p were identified by literature database searches.

Six mRNA targets of hsa-miR-132-3p (HN-1, RASA1, KLH-L11, MAPT, SPRED1 and ARHGAP32) and four mRNA targets of hsa-miR-503-5p (IGF1-R, FGF-R, L1CAM and CX3CL1) were chosen for further investigation based on their functional properties.

**Fig. 5:**
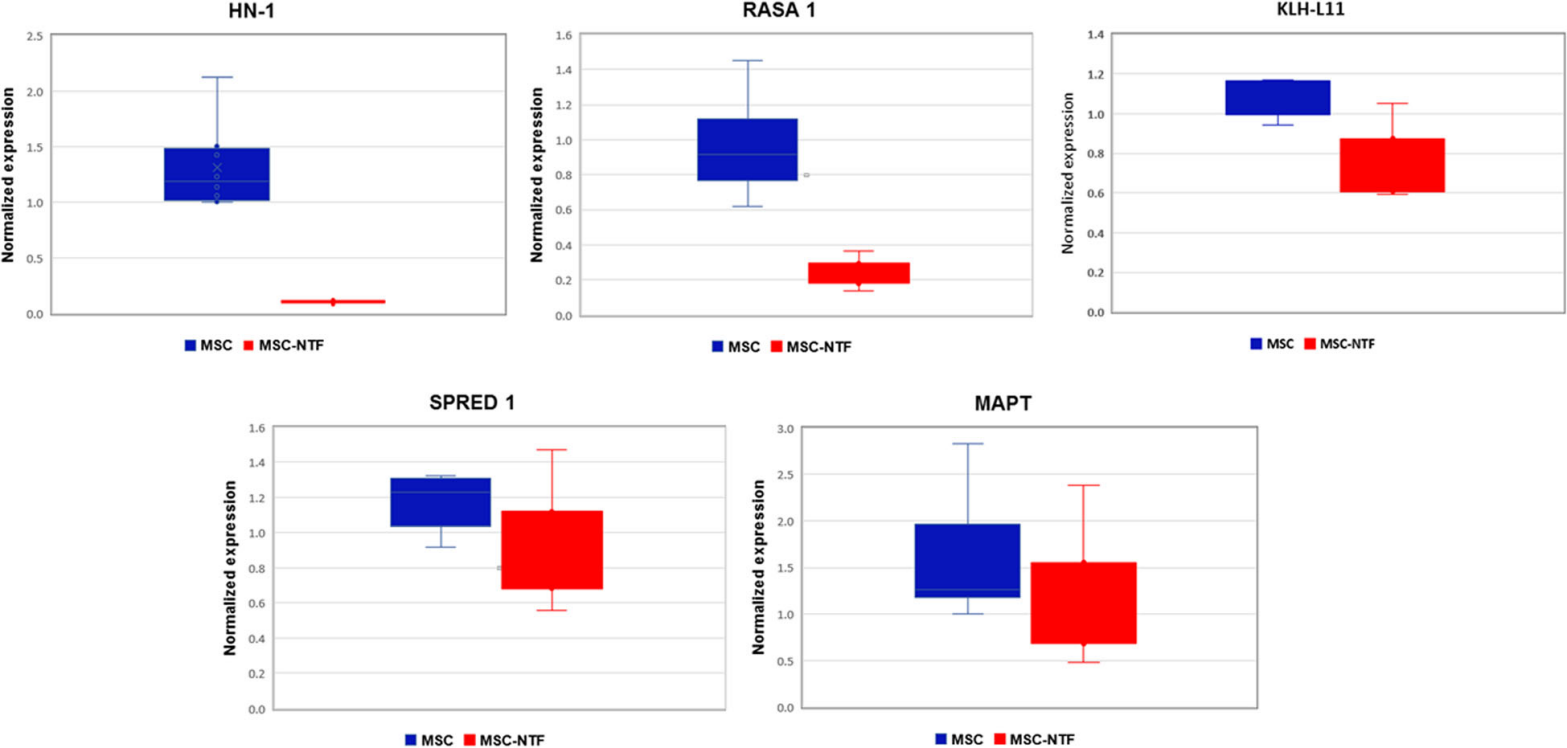
Differential expression of mRNAs targets of hsa-miR-132-3p. Expression of mRNA targets of miR-132-3p were compared in MSC and MSC-NTF cells of MSC and MSC-NTF cells of eight different donors (six ALS patients and two healthy donors) by qPCR analysis. HN-1, RASA1 and KLH-L11 were significantly downregulated (11.86 fold, 3.73 -fold and 1.5-fold respectively, *p* < 0.05). *MSC* mesenchymal stromal cells, *NTF* neurotrophic factors

Of the known mRNA targets of hsa-miR-132-3p that were analyzed, HN-1, RASA1, and KLH-L11 were found to be significantly downregulated 11.86-fold, 3.73-fold, and 1.5-fold, respectively. These mRNA targets were significantly downregulated in MSC-NTF cells compared to MSCs (*p* < 0.05, Fig. 5) while MAPT and SPRED1 were not found to be differentially expressed between the two cell types (Fig. 5).

Of the four mRNA targets of hsa-miR-503-5p, that were evaluated, expression of FGF-R and IGF1-R were found to be unchanged between MSC and MSC-NTF cells (data not shown). L1CAM and CX3CL1 were not expressed in either MSC nor in MSC-NTF cell samples (data not shown).

## Discussion

The results of this study suggest that the differentiation process of MSC-NTF cells leads to both an increased secretion of NTFs and a characteristic miRNA profile. Furthermore, individual miRNAs biomarkers can be used to characterize MSC-NTF cells and distinguish them from the MSCs of origin. The changes in miRNA expression following MSC-NTF differentiation appear to lead to a repression of pro-proliferative target genes (inducing cell cycle arrest) and anti-apoptotic genes, while inducing neuronal-related genes providing the molecular basis for the functional phenotype and putative mechanism of action of the differentiated cells.

Of the 160 miRNAs identified by the microarray analysis, hsa-miR-3663-3p was found to be the most strongly upregulated (Fig. 4) and confirmed by qRT-PCR to be strongly induced (18.5-fold). Microarray analysis also identified hsa-miR-132-3p, which was confirmed to be upregulated 4-fold, while upregulation of hsa-miR-150-3p could not be confirmed by qPCR.

The putative mRNAs targets of the miRNAs that were identified to be characteristic of MSC-NTF cells were also studied. The most strongly induced miRNA, miR-3663, has no known mRNA targets. We therefore focused our investigation on known mRNA targets of miR-132-3p and miR-503-5p.

We found the mRNA targets of hsa-miR-132-3p, HN-1 RASA1 and KLH-L11, to be significantly downregulated in MSC-NTF cells compared to MSC samples from the same donor (*p* < 0.05), while mRNA targets of miR-503-5p FGF-R and IGF1-R were unchanged.

Importantly, miR-320a, miR-424-5p and miR-503 which were downregulated in MSC-NTF cells, were previously reported to target VEGF [15–17]. Indeed, downregulation of these three miRNAs is correlated with a significant increase in VEGF secretion in MSC-NTF cells (Fig. 1), suggesting that the observed change in these microRNAs may contribute to the increase in VEGF expression. Further confirmatory studies will be required.

miR-132-3p, upregulated in MSC-NTF cells, is an evolutionarily conserved and neuron-enriched miRNA [18, 19] that has been shown to be a positive regulator of developing neuron axon extension, acting through repression of Rasa1 mRNA (a Ras GTPase activator), in a mechanism that operates locally within the axon [20].

The Hematopoietic- and neurologic-expressed sequence 1 (Hn1) gene encodes a small protein that is highly conserved among species. Hn1 has previously identified as a gene associated with nervous system development and nerve regeneration [21]. MiR-132 has been found to downregulate HN1 expression leading to reduced cell proliferation [22]. Depletion of Hn1 has been shown to result in cell cycle arrest, which is consistent with the differentiated phenotype of MSC-NTF cells [23].

Very little is known about the Kelch-like family member 11 (KLH-L11); however, other members of the Kelch-like family of genes are involved in a wide range of processes that are relevant to LTP, including inter/intracellular communication, cell morphology, cytoskeletal organization and protein binding [24].

## Conclusions

The results of this study suggest that a distinct miRNA signature may differentiate MSC-NTF cells from the MSCs of origin and potentially could be used as a sensitive criterion for identity -release testing in clinical trials. The upregulation of miR-132 supports a molecular mechanism for cell cycle arrest, neuronal differentiation and axonal extension, while downregulation of miR-320a, miR-424-5p and miR-503 are consistent with the observed increase in VEGF signaling. These observations support the putative mechanism of action of MSC-NTF cells and may have important implications to their application in ALS clinical studies and more broadly in a variety of neurodegenerative disorders that share common biology.
